# Modelling the Condensation Process of Low-Pressure Refrigerants in Mini-Channels

**DOI:** 10.3390/ma15134646

**Published:** 2022-07-01

**Authors:** Małgorzata Sikora, Tadeusz Bohdal

**Affiliations:** Department of Energy Engineering, Koszalin University of Technology, 75-459 Koszalin, Poland; tadeusz.bohdal@tu.koszalin.pl

**Keywords:** condensation, mini-channels, correlation, Novec649, HFE7100, HFE7000

## Abstract

The measure of the energy efficiency of the non-adiabatic two-phase condensation process of refrigerants in mini-channels is both the value of the heat transfer coefficient α and the flow resistance expressing the external energy input required to realize the flow. The modelling of this very complex process is effective if the condensation mechanism in mini-channels is correctly identified. It has been proven that the effects of changes in the condensation mechanism are the different structures of the two-phase flow resulting from process interactions both in the channel cross-section and along the flow path. The research aimed to connect the value of the heat transfer coefficient with the flow structures occurring during condensation. Thermal and visualization studies of the condensation process of low-pressure refrigerants were carried out: Novec649, HFE7100 and HFE7000 in tubular mini-channels with diameters d_h_ = 0.5; 0.8; 1.2; 2.0 mm. Based on visualization studies, flow structures were proposed to be divided into 3 main groups: dispersive, stratified and intermittent. Based on this, a computational correlation was derived for determining the heat transfer coefficient and frictional resistance depending on the type of flow structure. The research shows that the highest values of the heat transfer coefficient occur during the mist flow and the lowest during the bubble flow.

## 1. Introduction

Changes in the conditions of the condensation process have a direct impact on the formation of various mechanisms of momentum and energy exchange. These in turn are interdependent on the flow structures of two-phase flow condensation. It should be clearly emphasized that the conditions for the formation of two-phase condensation flow structures depend on the channel diameter. They are different in conventional channels than in mini-channels. Recognition of these conditions requires the use of visualization and thermal tests. In this case, the term two-phase flow structure is understood to mean the mutual configurations of the liquid and vapour phases in the channel cross-section. The combination of flow structures with heat transfer and flow resistance is of great importance in the design and operation of refrigeration and air conditioning heat exchangers. The flow structures during the condensation process are classified into three main groups: dispersed, intermittent and stratified. Dispersed flow is a structure in which the dispersed phase is in a continuous phase in the form of very smal gas bubbles (bubbly) or liquid drops (mist). Stratified flow is if the liquid flows in the lower part of the channel, while the gas phase is in the upper part, and the interface is smooth or there are small waves on it. Intermittent flow is if the amount of the liquid phase increases, the gas phase loses continuity and flows as bubbles and slugs separated by liquid plugs. The transition between the wave structure and the discontinuous one occurs with increasing wave height.

Mikielewicz et al. [1] investigated the flow resistance during condensation of HFE7000 and HFE7100 refrigerants in a mini-channel with a diameter of d = 2.3 mm. He compared these results with the correlations of such authors as Mishima and Hibiki (1996) [2], Lockhart and Martinelli (1949) [3], Fronk and Garimella (2010) [4], Del Col et al. (2011) [5] and Muller-Steinhagen and Heck (1986) [6]. The authors also proposed their correlation to determine the flow resistance. Mikielewicz et al. [7] also presented a model of the ring flow, which was created based on the results obtained from the literature for the boiling and condensation process of the refrigerants R404a, R600a, R290, R32, R134a, R1234yf. On this basis, a correlation was created describing the heat transfer coefficient and the pressure drops, presented in Table 1 and Table 2.

Table 1 shows selected correlations describing the pressure drop during condensation of refrigerants with low ODP (Ozone Depletion Potential) and GWP (Global Warming Potential). Table 2, on the other hand, contains selected correlations used to describe the heat transfer in the condensation process. These correlations are related to the structure that occurs during a two-phase flow.

Rahman et al. [11] performed research on the condensation of the R134a refrigerant in multiports made of finned and smooth channels with hydraulic diameters in the range d_h_ = 0.64–0.81 mm. The tests were performed at the saturation temperature T_s_ = 35 °C and G = 50–200 kg/m^2^s. In this publication, the correlations of many authors used to describe heat transfer were checked, including Bohdal et al. [16], Park et al. [17] and Shah [15]. Due to the insufficient compliance of the calculation results with the research results, the authors proposed their correlation. Del Col et al. [18,19] investigated the two-phase flows of natural refrigerants as (R290) and R1234ze in a mini-channel with a hydraulic diameter of d_h_ = 0.96 mm in the range of G = 100–1000 kg/m^2^s. The results of their research were compared with the models of Cavallini et al. [12] and Del Col et al. [8] and found the usefulness of these models in the described range.

**Figure 1:**
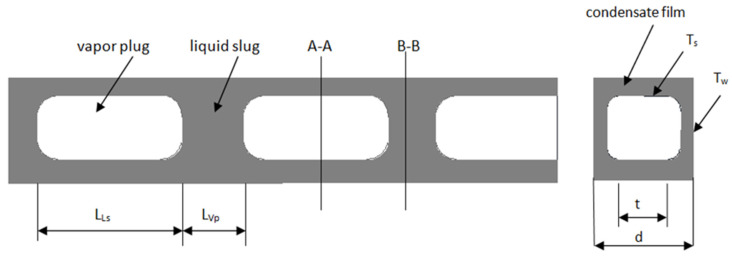
Heat transfer model for intermittent flow, where A-A and B-B are cross-section [9].

Jige et al. [9] described a heat transfer model for intermittent flow modifying the model for annular flow. The model concerns channels with a rectangular cross-section. These model assumptions are presented in Figure 1. As can be seen, the two-phase flow was divided into 2 parts, the vapour plug was treated as a part of the annular flow, and there was a liquid slug between the vapour plugs. On this basis, the correlation describing the Nusselt number for the intermittent flow was created. These equations are presented in Table 2.

Morrow et al. [20] tested the suitability of 12 correlations describing the condensation of refrigerants with low ODP and GWP. The comparison shows that the Cavallini et al. [21] and Kim and Mudawar [22] correlations were the best predictors for emerging synthetic refrigerants (e.g., R1234yf, R1234ze (E), R32, R450A, R513A, etc.). The Kim and Mudawar [22] correlation is recommended for propane and R600a; although Macdonald and Garimella [23] had the lowest MAE for CO_2_. Some of these correlations are presented in Table 2, including the Shah [15] correlation.

Wang et al. [10], made a non-equilibrium film model for condensing the low GWP refrigerant mixture R1234yf/R32 in a 4 mm diameter channel. Whereas Ko et al. [24] proposed a correlation to determine the Nusselt number during the condensation of the R123 factor in a plate heat exchanger.

As can be seen, in the literature can be found correlations describing the heat transfer coefficient depending on the flow structure. Unfortunately, upper described studies mainly concern medium and high-pressure refrigerants. They are rarely supported by visualization research. This work aims to present the dependence of the heat transfer coefficient and flow resistance on the type of the existing flow structure. An important aspect of this work is the use of new, pro-ecological, low-pressure refrigerants for research. The results of thermal tests are compared here with the results of observations of the condensation process in mini-channels. The authors published the results of flow structure and thermal studies during condensation of HFE7000, HFE7100 and Novec649 refrigerants in mini-channels [25,26,27]. Based on these studies, the mathematical model presented later in the article was created.

Nowadays, there has been a growing interest in the use of low-pressure refrigerants which have a low environmental impact. These types of refrigerants include HFE7000, HFE7100, HFE7200, HFE7300, FC-72, HFE-649 etc. All these refrigerants have a wide variety of thermal applications. Research on the use of the HFE-649 refrigerant in the Rankin cycle was carried out by Alshammari et al. [28], and a similar Rankin cycle study on the R245f fluid was carried out by Dong and Jeong [29]. Adebayo et al. [30] investigated the use of the new environmentally friendly refrigerants HFE7000 and HFE7100 in cascade systems in combination with CO_2_. The use of CO_2_ in refrigeration systems and heat pumps is also currently very much discussed in the literature. Piasecka and Strąk [31], on the other hand, performed boiling tests of the above-mentioned refrigerants with a zero GWP coefficient (FC-72, HFE-649, HFE-7000 and HFE-7100). These tests were carried out in rectangular mini-channels with a cross-section of 1.7 × 16 and 1 × 6.

As can be seen, the condensation process in mini-channels is complex, with many aspects that have not been sufficiently researched and described in the literature. Each flow phenomenon is accompanied by specific thermal phenomena and vice versa. So there are many aspects to consider during designing compact condensers.

## 2. Test Stand

**Figure 2:**
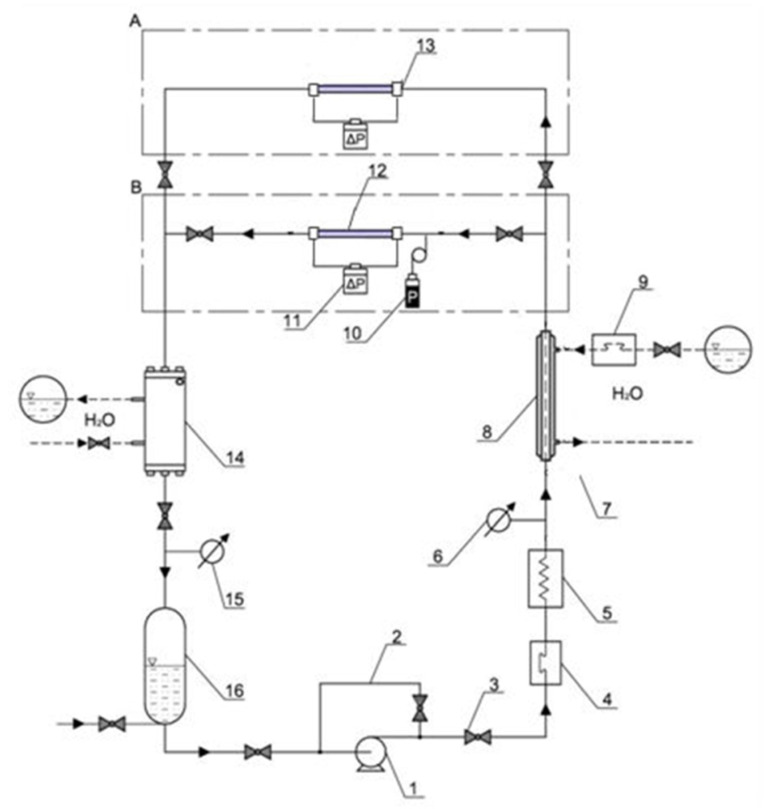
Schematic diagram of the test stand for thermal-flow and visualization tests of diabatic flow; A—section for thermal-flow tests of refrigerant condensation, B—section for visualization tests of two-phase flow structures; 1—ceramic pump, 2—bypass to regulate the flow rate, 3—valve, 4—electronic flowmeter of the refrigerant, 5—evaporator, 6—manometer, 7—temperature sensors (K-type thermocouples), 8—preliminary heat exchanger type “pipe in a pipe”, 9—electronic flow meter for measuring the water flow rate, 10—pressure sensor on the inlet to the measuring section, 11—pressure difference sensor, 12—horizontal section for flow visualization, 13—horizontal section for the thermal- flow investigations, 14—heat exchanger (sub-cooler), 15—manovacometer, 16—liquid refrigerant tank.

Investigations of the condensation process of low-pressure refrigerants HFE7000, HFE7100 and Novec649 were carried out on a specially designed test stand. On the stand, it was possible to simultaneously perform thermal-flow and visualization tests in the field of two-phase flow structures. Figure 2 shows a schematic diagram of the test stand. The test stand consisted of two measuring sections marked with symbols A and B (Figure 2). Section A was intended for the performance of experimental thermal-flow tests on the condensation of refrigerants in mini-channels. The measuring section 13 (Figure 2) was made of stainless steel. Section B is built in the form of a glass mini-channel used to visualize the two-phase flow of the refrigerant in the condensation process.

**Figure 3:**
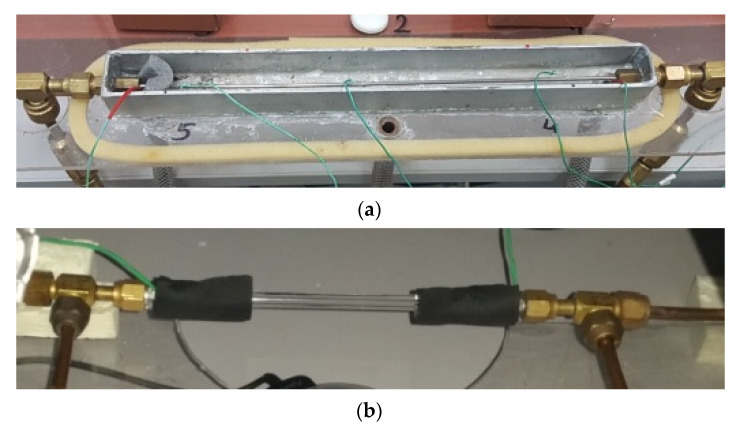
Overall view of the measuring sections: (**a**) for thermal investigations, (**b**) for visualisation.

In the system shown in Figure 2, the liquid refrigerant is sucked in by the ceramic pump 1 and forced to the heat exchanger in the form of a coil immersed in water tank 5, which functions as an evaporator. A system of electric heaters is used to supply heat to the water in which the coil with the flowing refrigerant is immersed. The refrigerant is heated until it evaporates. The temperature of the vapour leaving the evaporator is measured and kept constant by a thermostat. On the inlet of the liquid refrigerant to the exchanger 5, the Coriolis 34XIP67 type mass flow meter 4, made in class 0.5, was installed. At the inlet to measure sections 12 and 13, a preliminary water-cooled heat exchanger 8 is installed to regulate the vapour quality of the refrigerant vapour. From the heat balance of this exchanger, the value of the vapour quality x at the inlet to the measuring section was determined. The mini-channel was placed in a water channel with dimensions of 250 × 25 × 30 mm. The heat of condensation was collected by cooling water in this channel (Figure 3a). The length of the mini-channel in the measuring section was L = 250 mm. K-type temperature sensors for measuring the temperature of the outer surface of the channel were installed along the length of the mini-channel at a distance of 125 mm. The flow rate of the cooling water was measured with a flow meter 9. The pressure of the refrigerant in the measuring sections was measured with a piezoresistive sensor 10 with a PMP 131-A1401A1W transducer manufactured by Endress + Hauser with a measuring range of 0–40 MPa, and class 0.5. The pressure drop in the mini-channel was measured with a pressure difference sensor 11, with a Deltabar SPMP transducer with a measuring range of 0–1.5 MPa and an execution class of 0.075. In section A, the temperature of the outer wall of the mini-channel along its length was measured in 3 sections, and the temperature of the cooling water was measured at the inlet and outlet of the channel. In section B, the temperature of the medium at the inlet and outlet of the glass channel was measured. 7 K type thermocouples sensors (with a thermocouple diameter of 0.1 mm) were used for this purpose. All thermoelectric sensors, before their installation at the stand, were overhauled against a standard glass thermometer with a 0.1 °C unit. The refrigerant vapour quality was controlled by the cooling water flow rate. The so-called sub-cooler 14. The refrigerant liquid was then directed to the liquid tank. A bypass 2 with valve 3 is also installed to regulate the flow rate of the refrigerant.

In the case of section B, consisting of a glass mini-channel, besides the pressure and temperature sensors (the same as in section A), an Olympus i-SPEED 3 time-lapse camera with CMOS sensor was additionally installed, with a maximum recording speed of 10,000 fps and a maximum resolution of 1280 × 1024 pixels, along with software and data acquisition system. The research used the AF-S VR Micro-Nikkor 105 mm f/2.8 G IF-ED lens and the Nikkor AF 18–35 mm f/3.5–4.5 D IF-ED wide-angle lens from Nikon. Figure 3 show a view of both measuring sections.

**Figure 4:**
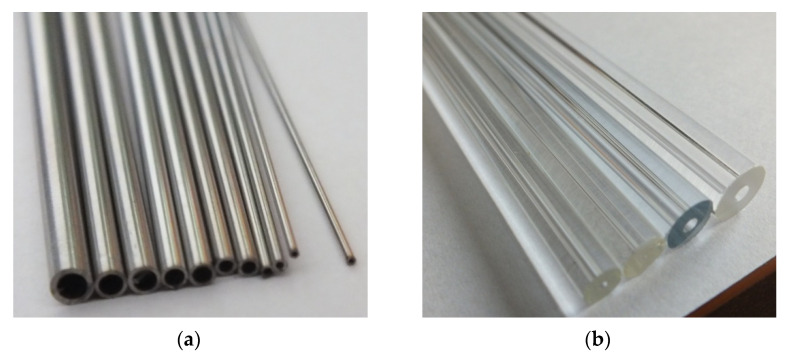
View of mini-channels (**a**) made of stainless steel-for thermal tests, (**b**) glass-for visualization tests.

The thermal-flow tests were carried out in stainless steel mini-channels, presented in Figure 4a. On the other hand, the visualisation tests were performed in the glass mini-channels presented in Figure 4b. In both cases, channels with a hydraulic diameter of d_h_ = 2 mm; 1.2 mm; 0.8 mm; 0.5 mm and a length of 250 mm were selected. This is due to the necessity to compare the results of thermal-flow tests with the visualization results. This is necessary to describe the range of occurrence of individual flow structures using thermal and flow parameters in the form of a flow structure map for the condensation process of low-pressure refrigerant in mini-channels [27,32,33].

In experimental tests, the following low-pressure refrigerants were used: HFE7000, HFE7100, Novec 649. The two-phase condensation process took place in the flow-through mini-channels with a hydraulic diameter: d_h_ = 2 mm; 1.2 mm; 0.8 mm and 0.5 mm. The tests were carried out in the ranges of changes in the characteristic condensation parameters:

- mass flux density: G = 180–5500 kg/(m^2^s),
- heat flux density: q = 0–200 kW/m^2^,
- saturation temperature: T_s_ = 30–70 °C,
- vapour quality: x = 1–0.

The selected refrigerants are low-pressure substances, which allows for the visualization of the condensation process in glass mini-channels. The advantage of these refrigerants its easy availability on the European market and low values of ODP and GWP coefficients, which means that they have a relatively low impact on environmental degradation. Unfortunately, low-pressure refrigerants are rarely used in classic refrigeration systems nowadays, but the results obtained for these refrigerants as standard, after being generalized, can be transferred to other, commonly used refrigerants.

**Figure 5:**
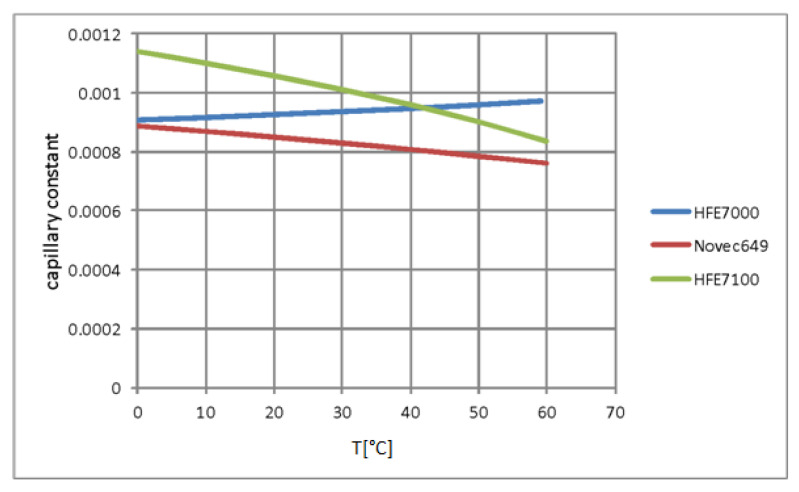
The dependence of the capillary constant of the investigated refrigerants on temperature.

Table 3 shows, for example, the properties of the HFE7000, HFE7100, and Novec649 refrigerants. Figure 5 shows the dependence of the capillary constant of all three fluids, described as ${\left(\frac{\sigma }{\left(\rho_{l}-\rho_{v}\right)g}\right)}^{0.5}$, on temperature. As can be seen, this relationship is different for each of the refrigerants, which is illustrated by the discrepancy in their physicochemical properties.

In section A of the test stand, the following parameters were directly measured:

(a) temperature of the medium along with the channel length T_r_,
(b) the pressure of the refrigerant at the inlet to channel p,
(c) the pressure drop along the length of the mini-channel Δp,
(d) cooling water temperature along with the channel length T_w_.

In section B, the following were measured:

- temperature of the refrigerant at the inlet and outlet from the measuring section t_r_,
- the pressure of the refrigerant at the inlet and outlet of the measuring section, p.

The refrigerant mass flow rate ṁ_r_ was also measured directly on the stand.

To determine the vapour quality of the refrigerant x at the inlet to the measuring section, the balance of the preliminary exchanger 8 was made (Figure 2). For this purpose, the temperature of the cooling water and the refrigerant was measured both on the inflow and outflow from the exchanger. The flow rate of cooling water through the exchanger 8 was also measured. The mass flux density G, the heat flux density on the inner wall of the mini-channel q, the vapour quality of the refrigerant x, and the heat transfer coefficient α during condensation of the refrigerant, were determined indirectly. The methodology for determining this calues has been described in detail in the article [35]. The amount of heat exchanged with the environment in the process of condensation of the refrigerant in mini-channels was determined using the method developed based on the concept presented in the [36]. 

The pressure drops were determined directly, so the measurement accuracy can be determined based on the range and measurement accuracy of the devices, and it was about 2%. The heat transfer coefficient was determined by the indirect method. The systematic error results result from an error in the measurement method, the accuracy of the used measuring instruments, and the used calculation methods. The observer’s error also influences the measurement results. The analysis also took into account the influence of random error. The most probable mean square error was about 10%.

## 3. Experimental Investigation Results

**Figure 6:**
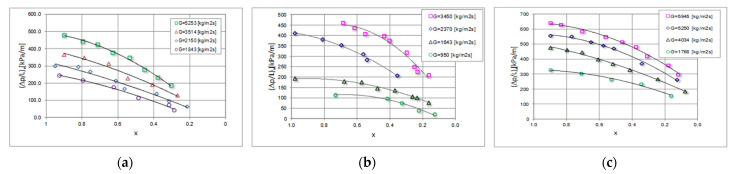
Experimental, local characteristics of pressure drops (Δp/L)_x_ depending on the vapour quality x during the condensation in a mini-channel with a hydraulic diameter d_h_ = 0.8 mm, refrigerants: (**a**) HFE7100; (**b**) Novec649; (**c**) HFE7000.

**Figure 7:**
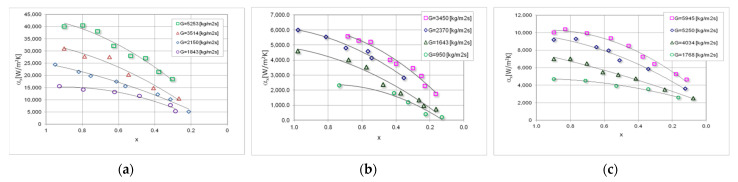
Experimental, local characteristics of heat transfer coefficient α_x_ depending on the vapour quality x during the condensation in a mini-channel with a hydraulic diameter d_h_ = 0.8 mm, refrigerant: (**a**) HFE7100; (**b**) Novec649; (**c**) HFE7000.

Figure 6 shows the dependence of the local pressure drops (Δp/l)_x_ on the local vapour quality x and the mass flux density G, for 4 mini-channels with different hydraulic diameters d_h_ = 2.0; 1.2; 0.8 and 0.5 mm, while Figure 7 shows the relationships describing the heat transfer coefficient α_x_, under the same process conditions. The comparison of the experimental, local characteristics of the pressure drops from the local value of the vapour quality x is presented in Figure 6, shows that in the specific condensation zone (x = 1–0) there is a decrease in the value of the local pressure drops (Δp/L)_x_. With a decrease in the local vapour quality, there is an increase in the content of the liquid phase in the entire volume of the flowing two-phase mixture. As can be seen, the highest pressure drops in the given conditions were obtained during the condensation of the HFE7000 refrigerant.

Figure 7 presents a summary of the experimental, local thermal characteristics of studied refrigerants condensation in mini-channels. It can be noticed that the increase in the vapour quality x and the mass flux density G have an analogous effect on the course of the characteristics as in Figure 6. In some cases, a slight increase in both, the local pressure drops (Δp/L)_x_ and the local heat transfer coefficient α_x_ can be observed, especially at the beginning of the condensation process, i.e., in the area x = 1–0.8. The highest values of the heat transfer coefficient occurred as seen during the condensation of the HFE7100 refrigerant.

**Figure 8:**
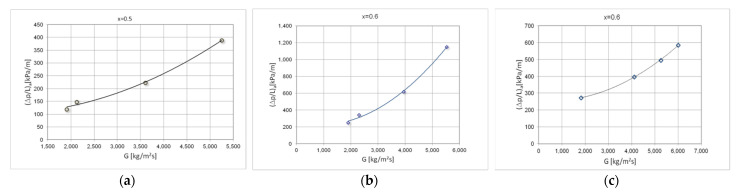
Experimental average flow characteristics of the pressure drop (Δp/L)_a_ depending on the mass flow density G during the condensation in a mini-channel with a hydraulic diameter d_h_ = 0.5 mm (T_s_ ≈ 50 °C), refrigerants: (**a**) HFE7100; (**b**) Novec649; (**c**) HFE7000.

**Figure 9:**
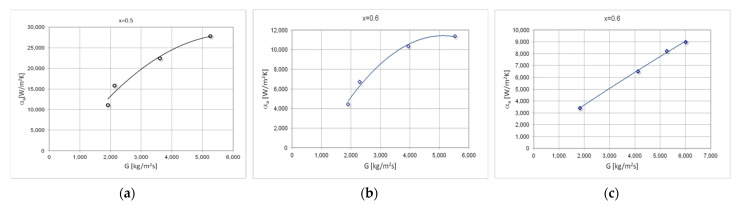
Experimental average flow characteristics of the heat transfer coefficient α_a_ depending on the mass flow density G during condensation in a mini-channel with a hydraulic diameter d_h_ = 0.5 mm (T_s_ ≈ 50 °C), refrigerants: (**a**) HFE7100; (**b**) Novec649; (**c**) HFE7000.

Figure 8 shows exemplary, experimental thermal-flow characteristics of the tested refrigerants condensation in the mini-channels, describing the average values of pressure drops (Δp/L)_a_. Figure 9 shows the average values of the heat transfer coefficient α_a_. The value of the average pressure drops and the average heat transfer coefficient were determined for the entire specific condensation zone. The characteristics of both diagrams show the effect of the mass flux density on both the pressure drops and the heat transfer coefficient. With the increase of the mass flux density G, the value of the mean pressure drops and the heat transfer coefficient increase.

## 4. Modelling of the Condensation Process in Pipe Mini-Channels

The measure of the energy efficiency of the condensation process of refrigerants in mini-channels is both the value of the heat transfer coefficient α and the pressure drops expressing the external energy input necessary to realize the flow. Modelling of this very complex process is effective when the correct identification of the condensation mechanism in the mini-channels is made [31]. During condensation in the flow, the mechanisms of mass, energy and momentum transfer change depending on the process parameters. It has also been proved that the effects of these changes are different structures of two-phase flow resulting from process interactions both in the channel cross-section and along the mini-channel. Among the characteristic parameters that influence the structure formation, the following can be mentioned: mass flux density G, a difference of the vapour and liquid phase velocity, phase density, vapour quality x, void fraction φ, where the nature of the flow can be defined by a dimensionless Reynolds number Re. If the above-mentioned parameters of the flow are known, then the structure of the two-phase flow (maps of flow structures) can be identified, and this allows to determine which elements of the momentum and energy transfer mechanisms are dominant. Therefore, mathematical modelling procedures can be applied to the recognized flow structure.

Extensive experimental research in the field of thermal and flow characteristics of condensation and visualization [32] of the two-phase flow structures allowed for the creation of a sufficiently wide database. On this basis, experimental data on the structures: dispersive, stratified and intermittent were distinguished. In the modelling process, over 500 experimental points were used for condensation of HFE7000, HFE7100, Novec649 refrigerants in mini-channels with hydraulic diameters d_h_ = 0.5; 0.8; 1.2 and 2.0 mm. The tests were carried out in the range of: mass flux density G = 180–5500 kg/m^2^s, vapour quality x = 1–0, and saturation temperature T_s_ = 30–70 °C.

Based on the theory of similarity and dimensional analysis, it was found that the formulation of a mathematical model capturing the results of experimental research can be given in the form of a functional relationship between dimensionless numbers. Therefore, the procedures of dimensional analysis were introduced, with known characteristic physical variables [33]. To determine the form of the equation linking the characteristic of condensation thermal and flow parameters and their relation to the existing flow structure, dimensional analysis was carried out based on π Buckingham’s theory. By applying the principles of dimensional analysis in turn, the following equation was obtained:

(1) $$Nu=M\cdot Re_{l}{}^{0.78}\cdot Pr_{l}{}^{C}$$

where *M* is the correlation-added value, a coefficient describing the effect of the refrigerant properties of the factor and the vapour quality, *Re_l_*—Reynolds number of liquid, *Pr_l_*—Prandtl number for liquid. A Nusselt number is a criterion number that describes the heat transfer process using convection and conduction. The Reynolds number is a criterion number that describes the type of flow (laminar, transitional, turbulent), it describes the ratio of inertia forces to viscous forces. The Prandtl number, on the other hand, combines the viscosity of a fluid with its thermal properties. Coefficient *M* is calculated from the equation:

(2) $$M=0.035\cdot {\left(\frac{\rho_{v}}{\rho_{l}}\right)}^{A}{\left(\frac{x}{1-x}\right)}^{0.77}p_{r}{}^{B}$$

where *ρ_v_* and *ρ_l_*, respectively, are the density of the vapour and liquid phases, *x*—vapour quality, *p_r_*—reduced pressure (being the ratio of saturation pressure p_s_ and critical pressure p_kr_), *A*, *B* and *C* are coefficients with dependent of the flow structures type. The presented form of the *M* parameter results from the properties of the investigated refrigerants, it takes into account the most important thermodynamic properties of refrigerants, influencing the heat transfer process. The selected form of the *M* parameter results from the literature review includes the most frequently used elements in the description of the condensation process in mini-channels. In this form, the parameter *M* allows to generalize the Equation (1) to use it for factors with significantly different thermodynamic properties.

To determine the unknowns in Equations (1) and (2), the Levenberg-Marquardt nonlinear regression model was used. The calculations were performed using standard procedures in the Statistica software package. Table 4 shows the values of the coefficients in Equations (1) and (2).

Due to the discrepancy in the physicochemical properties of the examined refrigerants, in particular, the density of the vapour phase, the correlation takes into account the ratio of the density of both phases and the reduced pressure, which depend on the type of refrigerant.

In Table 5 shows the applicability range of the relationship (2) in the form of the Reynolds number range, Prandtl number and reduced pressure p_r_.

The relationship describing the pressure drops of the condensation flow for the three main groups of structures was derived similarly. Using the Friedel method [34] and modifying the dependence of Bohdal et al. [28,35,36] the equation was obtained:

(3) $${\left(\frac{\Delta p}{l}\right)}_{TPF}={\left(\frac{\Delta p}{l}\right)}_{lo}\Phi_{lo}^{2}$$

where the modified two-phase multiplier is described as:

(4) $$\Phi_{lo}^{2}=2.5\cdot M^{C}\cdot \left[E^{0.2}+\frac{F^{1.9}\cdot H^{4}}{We^{1.24}}\right]$$

where the quantity *M* is determined by the Equation (3), while *E*, *F* and *H* are correction coefficients, and We are the Weber number. The value of the *C* coefficient in Equation (4) depends on the development degree of the interface. The averaged values of the *C* coefficient depending on the type of two-phase flow structure, as follows:

- *C* = 0.1 for the dispersion structure,
- *C* = 0.2 for a delaminated structure,
- *C* = −0.35 for a discontinuous structure.

The correction coefficients *E*, *F* and *H*, according to the Friedel correlation [34], are calculated according to the Formulas (5)–(7):

(5) $$E={\left(1-x\right)}^{2}+x^{2}\cdot \frac{\rho_{l}\cdot f_{vo}}{\rho_{v}\cdot f_{lo}},$$

(6) $$F=x^{0.78}\cdot {\left(1-x\right)}^{0.24},$$

(7) $$H={\left(\frac{\rho_{l}}{\rho_{v}}\right)}^{0.91}{\left(\frac{\mu_{v}}{\mu_{l}}\right)}^{0.19}{\left(1-\frac{\mu_{v}}{\mu_{l}}\right)}^{0.7},$$

where *f_lo_* and *f_vo_* are the friction coefficients determined for a single-phase flow in a smooth pipe, respectively, of the liquid or gas phase, from the Baroczy dependence [35], in the form:

(8) $$f_{x}=8\cdot {\left[{\left(\frac{8}{Re_{x}}\right)}^{12}+{\left\{{\left[2457\cdot ln{\left(\frac{Re_{x}}{7}\right)}^{0.9}\right]}^{16}+{\left(\frac{37530}{Re_{x}}\right)}^{16}\right\}}^{-1.5}\right]}^{\frac{1}{12}};$$

the subscript *x* = *vo* is used in the case of the calculation of the friction coefficient *f_vo_* and *x* = *lo* in the calculation of *f_lo_*. The Reynolds number Re for both cases is appropriately determined from the dependence:

(9) $$Re_{vo}=\frac{G\cdot d_{h}}{\mu_{v}},$$

(10) $$Re_{lo}=\frac{G\cdot d_{h}}{\mu_{l}},$$

Weber’s criterion number We is described as:

(11) $$We=\frac{d\cdot G^{2}}{\sigma \cdot \rho_{\mathrm{TPF}}},$$

where *ρ*_TPF_ is the density of the two-phase mixture described by the formula:

(12) $$\rho_{\mathrm{TPF}}={\left(\frac{x}{\rho_{v}}+\frac{1-x}{\rho_{l}}\right)}^{-1},$$

where: *x*—vapour quality, *μ*—dynamic viscosity coefficient, *ρ*—medium density (vapour and liquid phases, respectively), *G*—mass flux density. 

**Figure 10:**
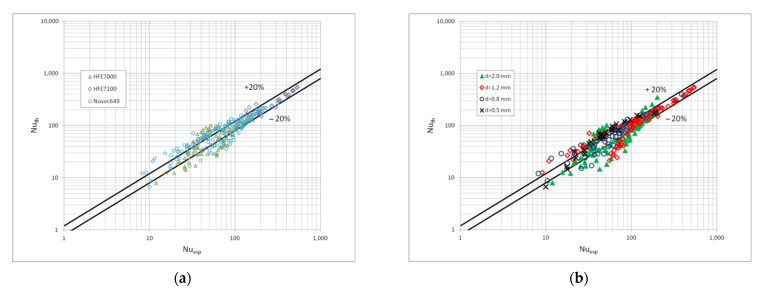
Comparison of the experimental investigation results of the HFE7000, HFE7100 and Novec649 refrigerants condensation in mini-channels which calculation results according to the correlation (1) for the dispersive structure; (**a**) for individual refrigerants, (**b**) for individual mini-channel diameters.

**Figure 11:**
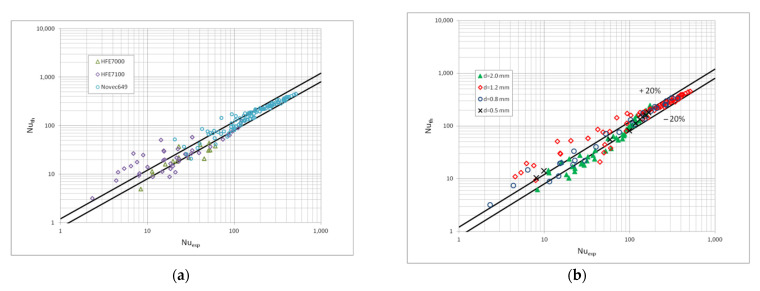
Comparison of the experimental investigation results of the HFE7000, HFE7100 and Novec649 refrigerants condensation in mini-channels which calculation according to the correlation (1) for the stratified flow; (**a**) for individual refrigerants, (**b**) for individual mini-channel diameters.

**Figure 12:**
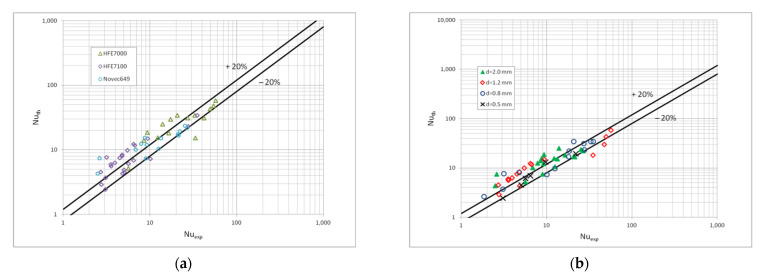
Comparison of the experimental investigation results of the HFE7000, HFE7100 and Novec649 refrigerants condensation in mini-channels which calculation according to the correlation (1) for the intermittent flow; (**a**) for individual refrigerants, (**b**) for individual mini-channel diameters.

Figure 10 shows a comparison of the heat transfer experimental studies results which the results of mathematical modelling according to the correlation (1) in terms of the dispersive structure, while Figure 11 and Figure 12 show the same comparisons for the stratified and intermittent structures. The above-mentioned figures also show the influence of the mini-channel diameter on the Nusselt number. As can be seen, the highest values of the Nusselt number were obtained for the mini-channel diameter d_h_ = 1.2 mm. It is mainly influenced by the parameters of the condensation process. 

**Figure 13:**
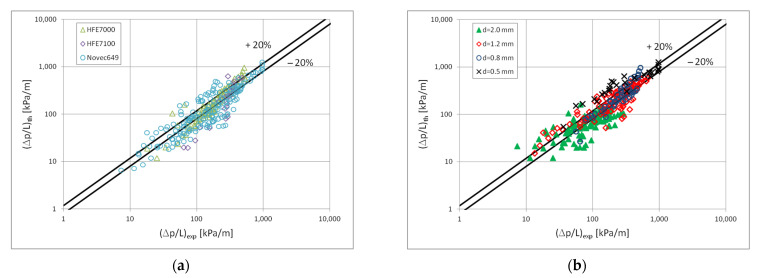
Comparison of the results of experimental studies on the condensation of the investigated refrigerants in mini-channels in terms of pressure drops with the proprietary correlation (3) for the dispersion structure; (**a**) for individual refrigerant, (**b**) for individual mini-channels diameters.

**Figure 14:**
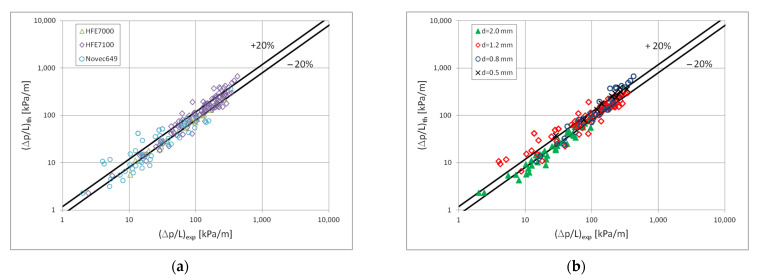
Comparison of the results of experimental studies of the investigated refrigerants condensation in mini-channels in terms of pressure drops with the correlation (3) for the stratified flow; (**a**) for individual factors, (**b**) for individual mini-channels diameters.

**Figure 15:**
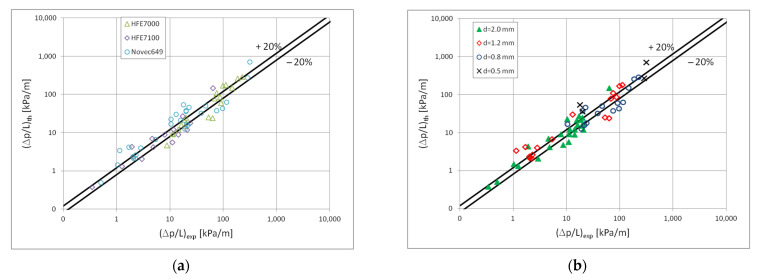
Comparison of the results of experimental studies of the investigated refrigerants condensation in mini-channels in terms of pressure drops with the correlation (3) for the intermittent flow; (**a**) for individual factors, (**b**) for individual mini-channels diameters.

Figure 13 presents a comparison of the results of the experimental tests of pressure drops with the results of calculations according to the correlation (3) and (4) in the scope of the dispersion structure. Figure 14 presents such a comparison for a stratified flow, while Figure 15 for a stratified flow. The comparison of the experimental results of the Novec649, HFE7000 and HFE7100 refrigerants condensation with the results of calculations according to the correlation (3) and (5) for all three two-phase flow structures (dispersive, stratified and intermittent) shows that 70% of the experimental points match at the level of ±20%. For nonadiabatic two-phase flows, the accuracy is satisfactory. Figure 13, Figure 14 and Figure 15 also show the influence of the mini-channel diameter on the flow resistance for each of the described groups of flow structures. It can be seen that the decrease in the hydraulic diameter of the mini-channel increases the pressure drops during the condensation process. A similar phenomenon was observed for all three groups of flow structures.

Table 6 and Table 7 show the statistical coefficients determined for the above-described models for the determination of the Nusselt number and the pressure drops of the condensation process in mini-channels, depending on the flow structure. These values were determined using the Statistica program. As can be seen, the regression coefficient is high in all cases. Only for the Nusselt number correlation determined for an intermittent flow that coefficient takes a value of about 0.82. This value is sufficient for thermal processes. The remaining parameters result from a wide range of tests.

**Figure 16:**
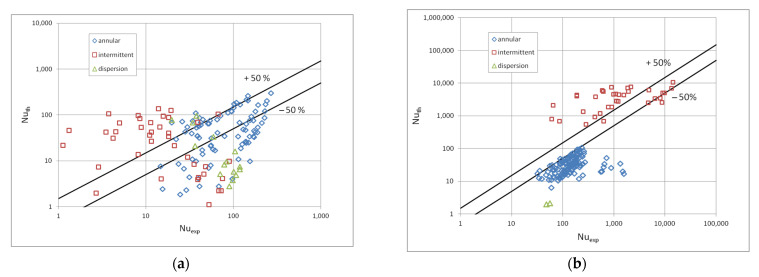
Comparison of the experimental condensation studies results of the refrigerant: (**a**) R134a, (**b**) R404a in mini-channels with calculations according to the author’s correlation (1) describing the heat transfer in terms of a stratified, intermittent and dispersive structure.

The proposed correlations (1) and (3) were additionally verified for the medium-pressure refrigerant R134a and high-pressure refrigerant R404A. The comparison of the results of the Authors experimental research on the condensation of the R134a refrigerant in mini-channels with the calculation results is shown in Figure 16a [5]. There is a large discrepancy between the results of the experiments and the results of calculations in the field of heat transfer. Only about 40% [5,8] of experimental points have an accuracy of 50%. In the case of comparing the pressure drops, there was a complete lack of adjustment of the calculation results with the results of experimental tests on the condensation of the R134a refrigerant. This means that the correlations (3) and (5) specified for the low-pressure refrigerants cannot be used in the calculation of the medium-pressure refrigerant R134a.

**Figure 17:**
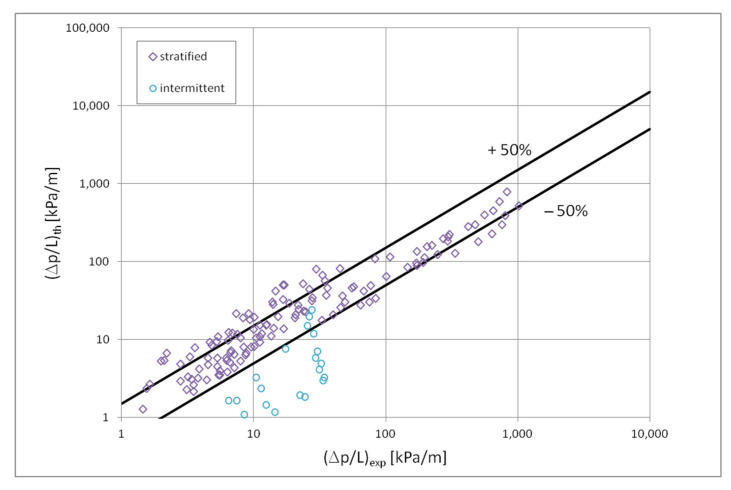
Comparison of the results of experimental research on the condensation of the R404A refrigerant in pipe mini-channels with the calculations according to the author’s correlation (3) describing the pressure drops in the scope of the stratified and intermittent flow.

Figure 16b and Figure 17 show a similar comparison of the results of experimental heat transfer and pressure drops tests during the condensation of the R404a refrigerant in mini-channels with the results of calculations according to the correlation (1) and (3), respectively. In the case of heat transfer, the discrepancy was too large, it is much higher in this case, as only about 10% of the results fall within the range of the discrepancy of ±50%. On the other hand, in the case of pressure drops the adjustment of the results of calculations and experimental tests for a stratified flow falls within the range of ±50%. It can be seen that this is a satisfactory accuracy, taking into account the divergence of the thermodynamic properties of the tested low-pressure refrigerant and the R404A refrigerant.

The heat transfer process is most intense during the dispersive structure. The appearance of a condensate film on the channel surface reduces the intensity of heat transfer. The thicker condensate layer gives the lower heat transfer coefficient. This is due to the thermal properties of the liquid phase. Therefore, discontinuous structures do not have a positive effect on the heat transfer process, and additionally, with small channel diameters and flow velocities, they may disturb the operation of the device. It is similar to the influence of structures on flow resistance. The dispersion structure gives higher flow resistance than the stratified and intermittent structures [25].

In other cases, a large discrepancy between the results of experimental investigations on the condensation of R134a and R404A refrigerants with the results of calculations according to the proposed correlations for low-pressure refrigerants is caused both by the difference in the physicochemical properties of the tested factors and the method of identifying flow structures during the condensation of R134a and R404A refrigerants. In this case, the calculation method was used to identify the two-phase flow structures based on the classification according to Cavallini et al. [9].

**Figure 18:**
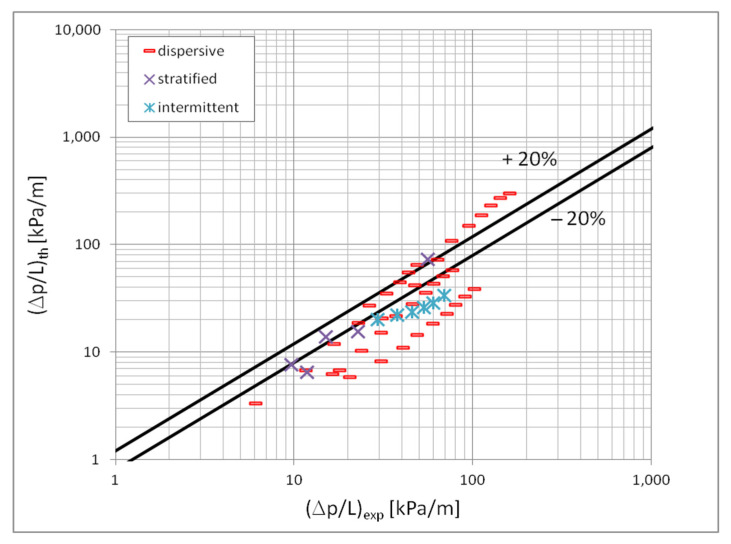
Comparison of the results of experimental research on pressure drop during condensation of HFE7000 and HFE7100 refrigerants according to Mikielewicz et al. [10] in a mini-channel d_h_ = 2.3 mm with calculations according to the correlation (3) in terms of dispersive, stratified and intermittent flow.

The results of the calculations according to the correlation (3) were also compared with the results of the research by Mikielewicz et al. [10] in terms of the pressure drops during condensation of HFE7000 and HFE7100 refrigerants in the pipe mini-channels d_h_ = 2.3 mm. The results of the comparison are shown in Figure 18. About 70% of the experimental test results were in agreement with the calculation results within 50%. This is due to a direct comparison of the values directly measured in experimental studies and those calculated according to the correlation. The identification of the structures was made with the use of the author’s map of flow structures.

## 5. Conclusions

The article presented the results of research on the condensation of HFE7000, HFE7100 and Novec649 refrigerants. The thermal tests were combined with the results of visualization tests on the condensation of these refrigerants published in Sikora [32]. Based on these studies, the correlation for the calculation of the Nusselt number and the pressure drops depending on the formed flow structure was presented. Based on the research, the following conclusions can be drawn:

1. During the condensation process in mini-channels, the highest pressure drops have HFE7000 refrigerant, while the highest values of the heat transfer coefficient have HFE7100. This is due to the physicochemical properties of these refrigerants.
2. Forming flow structures in the condensation process influences the intensity of heat transfer. The highest values of this coefficient are obtained in the dispersion flow and the lowest in the intermittent flow group. This is due to the increase in the thickness of the condensate layer, which acts as insulation on the inner surface of the channel.
3. Flow structures have a similar effect on the pressure drop value. This means that the highest flow resistance also results from the mist (dispersion) structure and the lowest for bubbly structure (intermittent flow).
4. As a result of the experimental research described in the article, a mathematical model was created to determine the pressure drops and the heat transfer coefficient during the condensation process in mini-channels, for individual groups of flow structures (dispersed, stratified and intermittent). This model is applicable for low-pressure refrigerants, in the scope of the parameters of the condensation process: mass flux density G = 180–5500 kg/(m^2^s), heat flux density q = 0–200 kW/m^2^, saturation temperature: T_s_ = 30–70 °C, vapour quality: x = 1–0.
5. The most difficult structure to analyze was the dispersion flow because it consists of very fine drops of liquid carried by the gas. Their size makes image analysis difficult, which causes a greater discrepancy between the test results and modelling results (Figure 10) compared to other structures.
6. The results of the calculations according to the new correlation were compared with the results of the experimental results of Mikielewicz et al. [10]. About 70% of the experimental test results were in agreement with the calculation results within 50%.

## Figures and Tables

**Table 1:** Correlation described pressure drops during the condensation process of new refrigerants for different flow regimes.

Author	Correlation	Comments
Mikielewicz et al. [7]	$\Phi_{TPC}^{2}=\Phi_{TPB}^{2}=\frac{f_{TPB}}{f_{0}}\;=\{\begin{array}{c} \Phi^{2}\left(1\pm \frac{B}{2}\right)for\;annular\;flow\\ \Phi^{2}\sqrt{1+\left(\frac{8\alpha_{PB}d}{\lambda RePrf_{0}\Phi^{2}}\right)}\;for\;other\;flow \end{array}$	Refrigerant: R404a, R600a, R290, R32,R134a, R1234yf,
Mikielewicz et al. [1]	$\Phi_{MS}^{2}=\left[1+2\left(\frac{1}{f_{l}}-1\right)\cdot x\cdot Con^{m}\right]\cdot {\left(1-x\right)}^{\frac{1}{3}}+\frac{1}{f_{l}}\cdot x^{3}$	d = 1.4–8 mm, refrigerant: R14ab, water, m = −1 for mini-channels, m = 0 for conventional channels
Del Col [8]	${\left(\frac{dp}{dz}\right)}_{f}=\Phi_{lo}^{2}\frac{2f_{lo}G^{2}}{d_{h}\rho_{l}}$	J_g_ > 2.5
Jige et al. [9]	${\left(\frac{dp}{dz}\right)}_{f}=\left[x^{1.8}+{\left(1-x\right)}^{1.8}\frac{\rho_{v}f_{lo}}{\rho_{l}f_{vo}}\;\;\;\;\;\;\;\;\;\;\;\;\;+0.65x^{0.68}{\left(1-x\right)}^{0.43}{\left(\frac{\mu_{l}}{\mu_{v}}\right)}^{1.25}{\left(\frac{\rho_{v}}{\rho_{l}}\right)}^{0.75}\right]\;\;\;\;\;\;\;\;\;\;\;\;\;\cdot \frac{2f_{vo}G^{2}}{d_{h}\rho_{v}}$	Intermittent flow
Wang et al. [10]	${\left(-\frac{dp}{dz}\right)}_{}=-G^{2}\frac{d}{dz}\left[\frac{x^{2}}{\varphi \rho_{v}}+\frac{{\left(1-x\right)}^{2}}{\left(1-\varphi \right)\rho_{l}}\right]\cdot {\left(-\frac{dp}{dz}\right)}_{f}$ ${\left(\frac{dp}{dz}\right)}_{f}=\Phi_{v}^{2}\frac{0.092G^{2}x^{2}}{\rho_{v}dRe_{v}^{0.2}}$ $\Phi_{v}=1+0.5\left[\frac{G}{\sqrt{gd\rho_{v}\left(\rho_{l}-\rho_{v}\right)}}\right]\cdot \chi_{tt}{}^{0.35}$	R1234yf/R32, d = 4 mm

**Table 2:** Correlation described heat transfer during the condensation process of new refrigerants for different flow regimes.

Author	Correlation	Comments
Mikielewicz et al. [7]	$\frac{\alpha_{TP}}{\alpha_{l}}=\sqrt{{\left(\Phi^{2}\right)}^{n}+\frac{C_{1}}{1+P_{1}}{\left(\frac{\alpha_{TP}}{\alpha_{l}}\right)}^{2}}$ $P_{1}=2.53\cdot 10^{-3}\cdot Re_{l}^{1.17}\cdot Bo^{0.6}\cdot {\left(\Phi^{2}-1\right)}^{-0.65}$	Refrigerant: R404a, R600a, R290, R32,R134a, R1234yf, For condensation C_1_ = 0, for boiling C_1_ = 1
Rahman et al. [11]	$Nu=2.271Re_{eq}^{1.226}Re_{lo}^{-0.667}Pr_{l}^{0.333}Bo_{eq}^{0.268}Ga^{0.497}$	Refrigerant R134a, d_h_ = 0.61–0.81 mm, smooth and micro-finned channels
Cavallini et al. [12]	$\alpha_{strat}=0.725{\left\{1+0.741{\left[\left(1-x\right)/x\right]}^{0.3321}\right\}}^{-1}\;\;\;\;\;\;\;\;\;\;\;\cdot {\left[\lambda_{l}^{3}\rho_{l}\left(\rho_{l}-\rho_{v}\right)gh_{lg}/\left(\mu_{l}d\Delta T_{SW}\right)\right]}^{0.25}\;\;\;\;\;\;\;\;\;\;\;+\left(1-x^{0.087}\right)\alpha_{lo}$	Stratified flow
Jige et al. [9]	$Nu=\varphi Nu_{An}+\left(1\;-\varphi \right)Nu_{Ls}$ $Nu_{An}={\left(Nu_{An\;F}{}^{3}+Nu_{An\;S}{\;}^{3}\right)}^{\frac{1}{3}}$	Intermittent flow
Hirose et al. [13]	$Nu={\left(Nu_{F}{}^{2}+Nu_{B}{\;}^{2}\right)}^{\frac{1}{2}}$ $Nu_{B}=1.60\eta^{-0.25}Bo^{-0.20}H\left(\varphi \right){\left(\frac{GaPr_{l}}{Ph_{l}}\right)}^{0.25}$ $\mathrm{wavy}\;\mathrm{flow}:\;Nu_{F}=7.85\sqrt{f_{v}}\left(\frac{\varphi_{x}}{\chi_{tt}}\right){\left(\frac{\mu_{l}}{\mu_{v}}\right)}^{0.1}{\left(\frac{x}{1-x}\right)}^{0.1}Re_{l}{}^{0.47}$ $\mathrm{annular}\;\mathrm{flow}:\;Nu_{F}=15.4\sqrt{f_{v}}\left(\frac{\varphi_{x}}{\chi_{tt}}\right){\left(\frac{\mu_{l}}{\mu_{v}}\right)}^{0.1}{\left(\frac{x}{1-x}\right)}^{0.1}Re_{l}{}^{0.43}$	Smooth and microfin channel with diameter d = 4 mm; refrigerants R32, R152a and R410A; G = 100–400 kg/m^2^s; T_s_ = 35 °C
Bashar et al. [14]	$Nu={\left(Nu_{F}{}^{2}+Nu_{B}{\;}^{2}\right)}^{\frac{1}{2}}$ $Nu_{F}=0.38\sqrt{f_{v}}\left(\frac{\varphi_{x}}{\chi_{tt}}\right){\left(\frac{\mu_{l}}{\mu_{v}}\right)}^{0.1}{\left(\frac{x}{1-x}\right)}^{0.1}Re_{l}{}^{0.9}$ $Nu_{B}=0.35Bo^{0.15}H\left(\varphi \right){\left(\frac{GaPr_{l}}{Ph_{l}}\right)}^{0.25}$	Refrigerant: R1234yf; d = 2–8 mm; G = 50–400 kg/m^2^s;
Shah [15]	Regime I *α* = *α*_1_ Regime II *α* = *α*_1_ + *α*_2_ Regime III *α* = *α*_2_ $\alpha_{1}=\alpha_{lo}\left[1+1.128x^{0.817}{\left(\frac{\rho_{l}}{\rho_{v}}\right)}^{0.3685}{\left(\frac{\mu_{l}}{\mu_{v}}\right)}^{0.2363}{\left(1-\frac{\mu_{v}}{\mu_{l}}\right)}^{2.144}Pr_{l}{}^{-0.1}\right]$ $\alpha_{2}=1.32Re_{ls}{}^{-\frac{1}{3}}{\left[\frac{\rho_{l}\left(\rho_{l}-\rho_{v}\right)g\lambda_{l}{}^{3}}{\mu_{l}{}^{2}}\right]}^{\frac{1}{3}}$	Refrigerant: butane, CO2, FC-72, propane, R1234fa, R1234ze, R134q, R152a, R22, R245fa, R32, R410A, water; d = 0.1–2.8 mm; G = 20–1400 kg/m^2^s

**Table 3:** Examples of the tested refrigerants properties at ambient pressure [34].

Quantity	Unit	HFE7000	HFE7100	Novec649
ODP	[-]	0	0	0
GWP	[-]	530	320	1
Molar mass	[kg/kmol]	200	250	316
Evaporation temperature	[°C]	34	61	49
Freezing point	[°C]	−122.5	−135	−108
Liquid density	[kg/m^3^]	1400	1520	1600
Surface tension	[N/m]	0.0124	0.0136	0.0108
Vapour pressure	[kPa]	64.6	26.93	40

**Table 4:** The value of the coefficients A, B and C in the Equations (1) and (2).

Coefficient	Two-Phase Flow Structure		
	Dispersive	Stratified	Intermittent
A	0.39	0.27	−0.78
B	−0.375	−0.23	−1.67
C	1.31	1.39	−2.74

**Table 5:** Range of Reynolds number Re, Prandtl number Pr and reduced pressure pr values in which the correlation was checked (1).

Quantity	Two-Phase Flow Structure		
	Dispersive	Stratified	Intermittent
Re	101–10,061	318–10,530	187–12,033
Pr	4.8–11.32	5.1–11.2	5.16–11.3
p_r_	0.02–0.094	0.025–2.073	0.035–0.071

**Table 6:** Statistical coefficients determined for the heat transfer model described by Equation (3).

Statistical Coefficients	Two-Phase Flow Structure		
	Dispersive	Stratified	Intermittent
Regression coefficient R.	0.91	0.92	0.82
Standard deviation	91.52	120.8	13.45
The confidence interval of the average	87.89 ÷ 109	99.69 ÷ 136	11.9 ÷ 18.47

**Table 7:** Statistical coefficients determined for the model for determining the two-phase flow resistance according to the Equations (5) and (6).

Statistical Coefficients	Two-Phase Flow Structure		
	Dispersive	Stratified	Intermittent
Regression coefficient R.	0.95	0.92	0.94
Standard deviation	390,802	144,208	26,983
The confidence interval of the average	252 × 10^3^ ÷ 342 × 10^3^	88 × 10^3^ ÷ 131 × 10^3^	62 × 10^3^ ÷ 170 × 10^3^

## Data Availability

The data presented in this study are available on request from the corresponding author.

## Nomenclature

A, B, E, F, H, M	coefficient
C	constant
Bo	Bond number,
Con	confinement number
d	diameter, m
dp/dz	frictional pressure drop, Pa/m
f	friction factor
g	acceleration due to gravity, m/s^2^
G	mass flux density, kg/m^2^s
Ga	Galileo number,
h_lg_	latent heat, J/kg
m	coefficient,
Nu	Nusselt number,
p	pressure, Pa
Ph	phase change number,
Pr	Prandtl number,
q	heat flux density, W/m^2^
Re	Reynolds number,
t, T	temperature, °C, K
We	Weber number,
x	vapour quality,
Δp/l	pressure drops, Pa/m
**Greek**	
α	heat transfer coefficient, W/m^2^ K
Δ	difference,
η	area magnifying ratio,
j	void fraction,
λ	conductivity coefficient, W/m·K
μ	dynamic viscosity, Pa·s
r	density, kg/m^3^
σ	surface tension, N/m
Φ^2^	two-phase multiplier
Χ_tt_	dimensionless two-phase flow multiplier Martinelli,
**Subscripts**	
a	average,
An	annular,
B	free convection,
eq	equivalent,
exp	experimental,
F	forced convection,
f	frictional,
h	hydraulic,
l	liquid,
lo	liquid only,
Ls	liquid slug,
MS	from Muller-Steinhagen and Heck model,
0	referencing case,
PB	pool boiling,
r	reduced, refrigerant
S	surface tension dominant
s	saturated,
v	vapour,
v_o_	vapour only,
th	theoretical,
tp	two-phase,
TPC	two-phase condensation
TPB	two-phase boiling
TPF	two-phase flow
w	water,
x	local
